# Semen enhances transmitted/founder HIV-1 infection and only marginally reduces antiviral activity of broadly neutralizing antibodies

**DOI:** 10.1128/jvi.01190-23

**Published:** 2024-03-19

**Authors:** Pascal von Maltitz, Lukas Wettstein, Tatjana Weil, Philipp Schommers, Florian Klein, Jan Münch

**Affiliations:** 1 Institute of Molecular Virology, University Ulm Medical Center, Ulm, Germany; 2 Laboratory of Experimental Immunology, Institute of Virology, University of Cologne, Faculty of Medicine and University Hospital of Cologne, Cologne, Germany; 3 German Center for Infection Research, Partner site Bonn-Cologne, Cologne, Germany; 4 Center for Molecular Medicine Cologne (CMMC), Cologne, Germany; 5 Department I of Internal Medicine, Faculty of Medicine and University Hospital of Cologne, University of Cologne, Cologne, Germany; Icahn School of Medicine at Mount Sinai, New York, New York, USA

**Keywords:** bNAb, HIV-1, semen, microbicide

## Abstract

Topically applied microbicides may play a critical role in preventing sexual transmission of human immunodeficiency virus type 1 (HIV-1); however, their efficacy can be compromised by amyloid fibrils present in semen, which significantly increase HIV-1 infectivity. This phenomenon may have contributed to the failure of most microbicide candidates in clinical settings. Understanding the impact of semen on microbicide effectiveness is thus crucial. In our study, we evaluated the influence of semen on the neutralizing activity of broadly neutralizing antibodies (bNAbs), including PG16, PGT121, 10–1074, 3BNC117, and VRC01, which are potential microbicide candidates. We found that semen enhances infection of HIV-1 transmitted/founder viruses but only marginally affects the neutralizing activity of tested antibodies, suggesting their potential for microbicide application. Our findings underscore the need to consider semen-mediated enhancement when evaluating and developing microbicides and highlight the potential of incorporating HIV-1 bNAbs in formulations to enhance efficacy and mitigate HIV-1 transmission during sexual encounters.

**IMPORTANCE:**

This study examined the impact of semen on the development of microbicides, substances used to prevent the transmission of HIV-1 during sexual activity. Semen contains certain components that can render the virus more infectious, posing a challenge to microbicide effectiveness. Researchers specifically investigated the effect of semen on a group of powerful antibodies called broadly neutralizing antibodies, which can neutralize a large spectrum of different HIV-1 variants. The results revealed that semen only had a minimal effect on the antibodies' ability to neutralize the virus. This is promising because it suggests that these antibodies could still be effective in microbicides, even in the presence of semen. Understanding this interaction is crucial for developing better strategies to prevent HIV-1 transmission. By incorporating the knowledge gained from this study, scientists can now focus on creating microbicides that consider the impact of semen, bringing us closer to more effective prevention methods.

## INTRODUCTION

The lack of a protective vaccine against the human immunodeficiency virus type 1 (HIV-1) fueled the search for effective prevention and therapy of HIV-1 infection. Although highly active antiretroviral therapy (ART) suppresses HIV-1 replication in patients, 1,500,000 new HIV-1 infections and 650,000 HIV-1-related deaths were registered by the World Health Organization (WHO) in 2021 (1). Since the majority of HIV-1 infections result from sexual transmission (2), microbicides pose a potential measure to avert HIV-1 infection. Microbicides are topically applied compounds that curb or prevent the spread of sexually transmitted diseases, for example, by shielding mucosal surfaces from invading pathogens, by directly inactivating them, or by interfering with their replication (3). Although microbicides effectively restrict HIV-1 infection or replication *in vitro*, clinical trials were unable to demonstrate the efficacy of polyanionic or nucleotide reverse transcriptase inhibitors in preventing or reducing HIV-1-acquisition (4–6). We previously reported that semen—the main vector for HIV-1 transmission (7)—contains cationic peptide fibrils derived from abundant seminal proteins that enhance the infectivity of HIV-1 (8–12). This infection enhancement considerably reduces the efficacy of microbicides such as anionic polymers, antibodies, or reverse transcriptase and integrase inhibitors, potentially explaining the conflicting results from *in vitro* studies and clinical trials on microbicides (13). The majority of anti-HIV-1 antibodies that are developed during infection exhibit no or very limited neutralizing activity against a broader range of HIV-1 variants and are therefore not broadly neutralizing (14). However, about 1%–5% of chronically infected individuals develop anti-HIV-1 antibodies of outstanding breadth and potency (15). Numerous studies explore the potential of these broadly neutralizing antibodies (bNAbs) for usage in HIV-1 prevention and therapy. Here, we investigated the potential use of highly potent HIV-1 bNAbs as microbicides in the presence of semen.

## RESULTS

**Fig 1:**
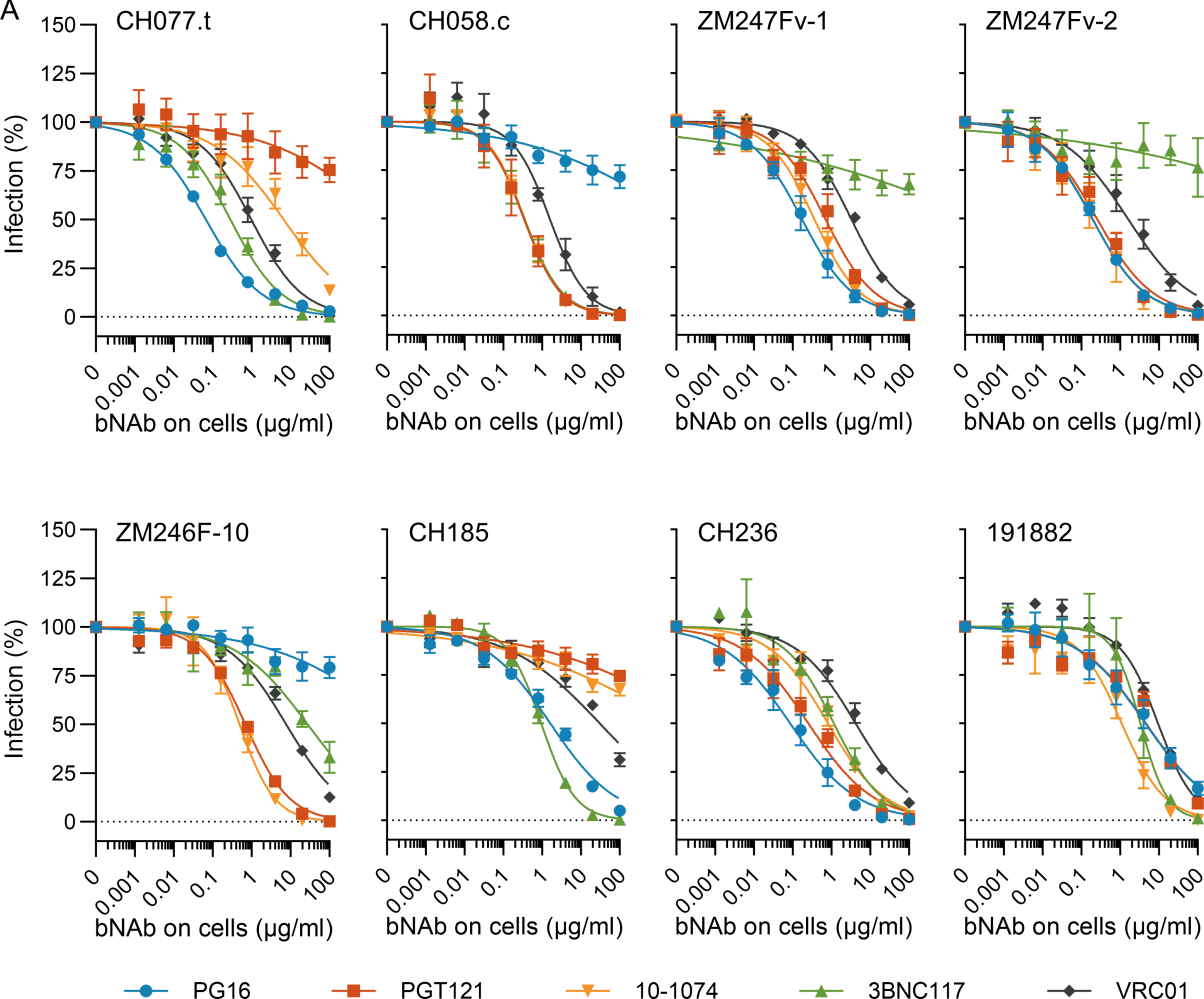
Broadly neutralizing antibodies inhibit HIV-1 transmitted/founder virus infection. TZM-bl cells were incubated with serial dilutions of different broadly neutralizing antibodies and infected with replication-competent HIV-1 transmitted/founder (T/F) strains. Infection rates were assessed at 2 dpi by measuring ß-galactosidase activity of cell lysates and were normalized to mock-treated cells. Shown are the mean values from *n* = 3 independent experiments ± SEM.

HIV-1 infection as a consequence of sexual transmission is predominantly established by a single virion, referred to as transmitted/founder (T/F) virus (2). In order to mimic sexual transmission more closely, we employed a panel of replication-competent HIV-1 T/F infectious molecular clones (IMC) to address the suitability of bNAbs as microbicides. First, we determined the susceptibility of eight HIV-1 T/F infectious molecular clones (IMC) toward a panel of bNAbs targeting different epitopes on the HIV-1 glycoprotein gp120: PG16 (V1/V2 loop) (16), PGT121 (17), and 10–1074 (V3 stem) (18), as well as 3BNC117 (19) and VRC01 (CD4 binding site) (20). As expected, all bNAbs were highly active against the majority of HIV-1 T/F IMCs with IC_50_s between 0.07 and 30.8 µg/mL; however, most T/F strains displayed resistance toward one or more bNAbs (Fig. 1; Table 1). All bNAbs neutralized T/F IMCs CH236 and 191882, whereas CH185 was susceptible only to PG16, 3BNC117, and – to a lesser extent – VRC01. T/F IMCs CH077.t, CH058.c, ZM247Fv-1, ZM247Fv-2, and ZM246F-10 were inhibited by four of the five tested bNAbs. Notably, PGT121 did not neutralize CH077.t, PG16 was ineffective against CH058.c and ZM246F-10, and 3BNC117 failed to inhibit ZM247Fv-1 and ZM247Fv-2 infection (Fig. 1; Table 1).

**TABLE 1:** IC_50_ (µg/mL) of bNAbs against HIV-1 transmitted/founder strains^*a*^

HIV-1 T/F virus	IC_50_ (µg/mL) of bNAb gp120 epitope				
	V1/V2 loop	V3 stem		CD4bs	
	PG16	PGT121	10–1074	3BNC117	VRC01
Clade B					
CH077.t	0.07	>100	6.7	0.3	1.0
CH058.c	>100	0.3	0.4	0.3	1.6
Clade C					
ZM247Fv-1	0.2	0.7	0.4	>100	3.2
ZM247Fv-2	0.2	0.3	0.2	>100	1.6
ZM246F-10	>100	0.7	0.5	27.7	7.8
CH185	1.6	>100	>100	1.0	30.8
CH236	0.1	0.3	0.8	1.2	4.2
Clade D					
191882	4.5	4.7	1.1	3.3	9.5

^*a*^ >100: no half-maximal inhibition at maximum compound concentrations observed; CD4bs: CD4 binding site.

**Fig 2:**
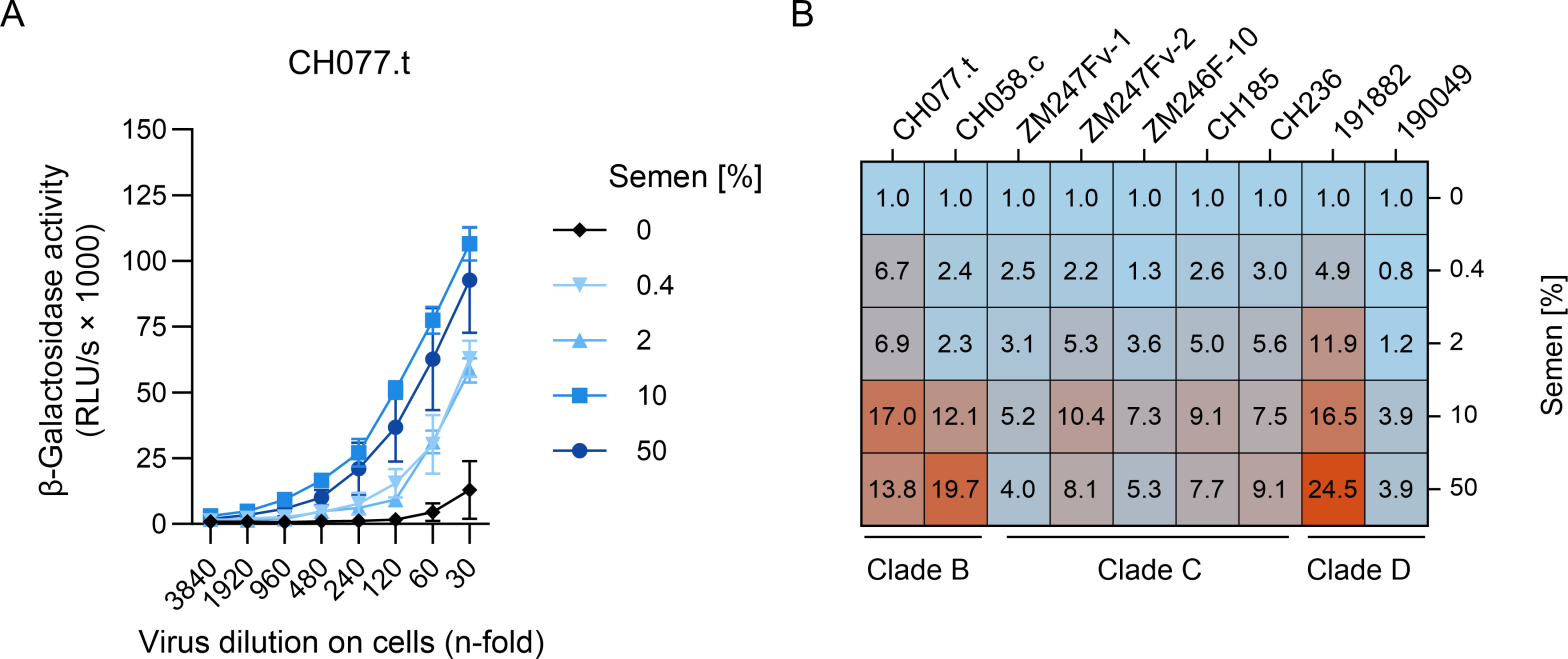
Semen enhances HIV-1 transmitted/founder infection. (**A**) Infection rates of TZM-bl cells infected with serial dilutions of semen (SE)-exposed (50%, 10%, 2%, and 0.4%) or PBS-treated (0%) replication-competent HIV-1 transmitted founder (T/F) CH077.t were assessed at 2 dpi by measuring ß-galactosidase activity of cell lysates. (**B**) Fold infectivity enhancement of nine SE-exposed (50%, 10%, 2%, and 0.4%) replication-competent HIV-1 T/F strains normalized to infectivity in the absence of semen yielding ß-galactosidase activity of 5,000–10,000 RLU/s. A-C show mean values from triplicate infections ± SD.

Next, we determined the effect of semen on HIV-1 T/F virus infection. To accomplish this, we mixed infectivity-normalized amounts of the viruses with various semen concentrations (0%, 0.4%, 2%, 10%, and 50% vol/vol) prior to infecting TZM-bl reporter cells, as described (13). Our findings reveal that semen significantly enhances the infection rates of HIV-1 T/F viruses, with concentrations of 10% and 50% vol/vol showing the most pronounced effect. This enhancement is depicted for HIV-1 CH077.t in Fig. 2A and summarized for all other tested viruses in Fig. 2B and Fig. S1, corroborating and expanding upon prior studies (8, 13).

In the absence of semen, the baseline infection rates were observed to be between 5,000 and 10,000 Relative Light Units (RLU) per second. Notably, the addition of 10% vol/vol semen resulted in a 4-fold to 17-fold increase in these rates (Fig. 2B). These data establish a reliable baseline for detecting infection in the absence of semen and demonstrate a significant augmentation of infectivity in its presence. However, treatment of virions with 50% vol/vol semen led to a cell culture concentration of 3.3%, which was associated with cytotoxic effects, as previously shown (13, 21–23). Consequently, a semen concentration of 10% vol/vol was selected for further investigations into the impact of semen on the activity of bNAbs, aligning with previous research evaluating the antiviral efficacy of candidate microbicides against HIV-1 (13).

**Fig 3:**
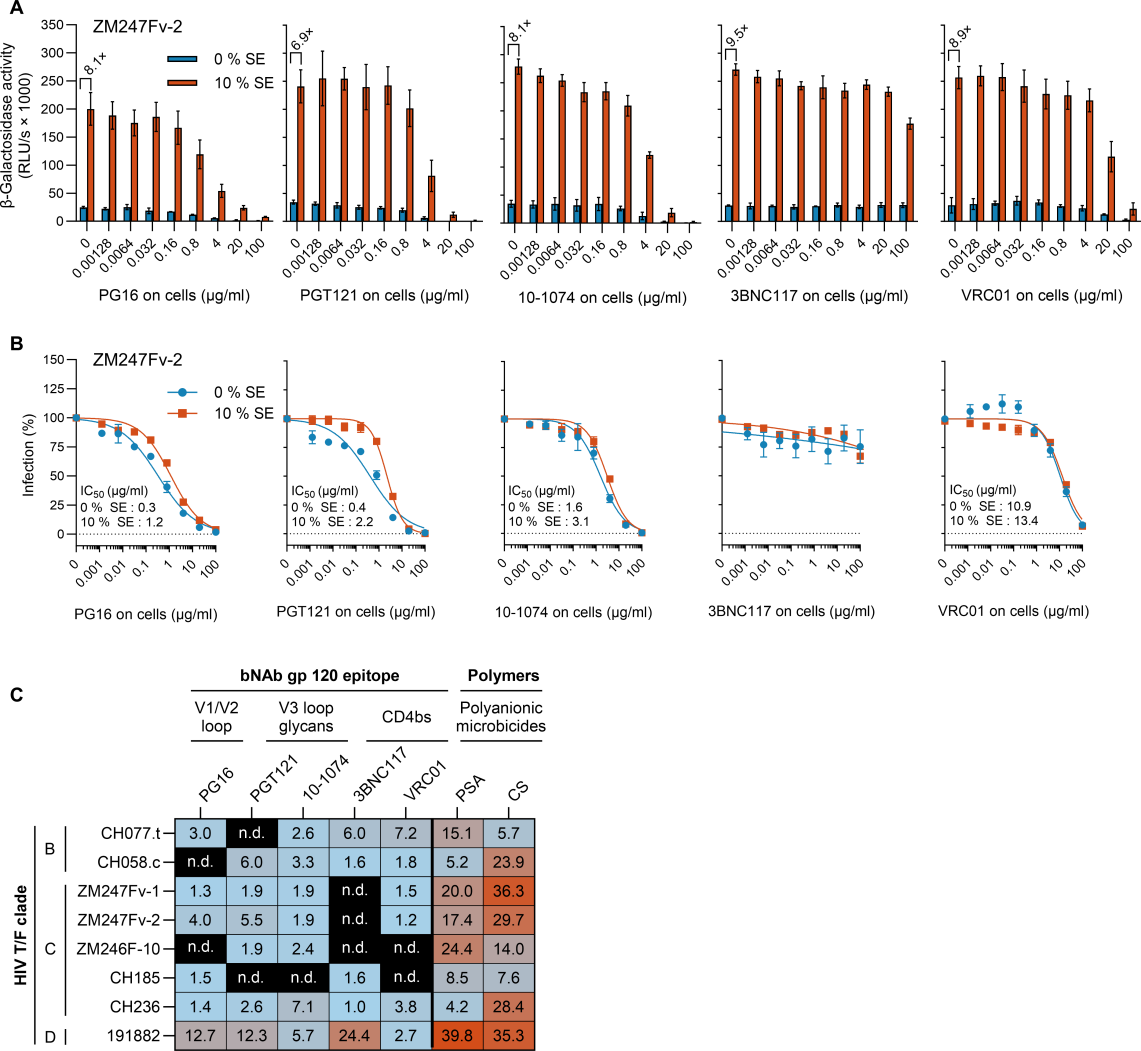
Inhibition of HIV-1 transmitted/founder ZM247Fv-2 by bNAbs is moderately reduced in the presence of semen. TZM-bl cells treated with serial dilutions of broadly neutralizing antibodies (bNAbs) and infected with semen- (SE) or PBS-treated HIV-1 T/F strain ZM247Fv-2. At 2 hpi, supernatants were removed, cells were washed, and a fresh medium supplemented with bNAb was added. Infection rates were assessed at 2 dpi by measuring ß-galactosidase activity of cell lysates and were normalized to mock-treated cells. (**A**) Infection rates of one representative experiment, in triplicates ± SD. Numbers indicate the fold-change of infectivity upon incubation with SE. (**B**) Normalized infection rates of *n* = 3 independent experiments, each in triplicates ± SEM. (**C**) Fold-change of inhibitory activity (IC_50_) of bNAbs and microbicides against semen-treated and mock-treated HIV-1 T/F IMCs. CD4bs: CD4 binding side; n.d.: IC_50_ not determined, since maximum inhibition >50%. For corresponding neutralization curves, refer to B and Fig. S2 to S4.

To this end, TZM-bl cells were exposed to serial dilutions of bNAbs before being infected with either semen (10% vol/vol) or PBS-treated HIV-1 T/F IMCs. As exemplarily shown for HIV-1 T/F ZM247Fv 2, semen exposure led to an average 8.3-fold (6.9-fold to 9.5-fold) increase in infection (Fig. 3A). The presence of semen slightly altered the effectiveness of bNAbs PG-16, PGT121, 10–1074, and VRC01, with changes in neutralizing activity ranging from 1.2-fold to 5.5-fold. Nonetheless, complete neutralization was achieved with antibody concentrations of 100 µg/mL, unaffected by semen addition (Fig. 3A and B). Conversely, semen significantly reduced the antiviral efficacy of microbicides polystyrene sulfonic acid (PSA) and cellulose sulfate (CS) against this virus, decreasing their potency by 17.4-fold and 29.7-fold, respectively (Fig. 3C; Fig. S2 and S3). Similar patterns were observed across most tested T/F viruses and bNAbs/polyanions, with bNAbs' IC_50_ values slightly increasing (1-fold to 7.2-fold) in the presence of semen. In contrast, IC_50_ values for PSA and CS rose substantially (4.2-fold to 39.8-fold and 5.7-fold to 36.3-fold, respectively) with semen. A standout was HIV-1 T/F 191882, a clade D virus, which showed a 2.7-fold to 24.4-fold decrease in neutralization susceptibility to bNAbs when incubated with semen.

We further investigated the influence of semen on the infection of HIV-1 T/F virus and its neutralization by bNAbs in primary CD4+ T cells, key target cells for HIV (13). This analysis paralleled the methodology used in TZM-bl cell assays, necessitating infection rates adequate to evaluate both the neutralizing capacity of bNAbs and the HIV-1 enhancing properties of semen. Initial tests with activated peripheral blood mononuclear cells (PBMCs) from various donors indicated only minimal infection rates by most T/F strains, thereby limiting the scope for a normalized and extensive analysis across all non-neutralizing antibodies (nNAbs) and T/F viruses. Consequently, we concentrated on the HIV-1 T/F strain CH077.t, which consistently showed detectable infection in CD4+ T cells (Fig. S6), to assess its response to semen and bNAbs. Virion incubation with 10% semen in fact increased the infection of CD4+ T cells by 3.1-fold (Fig. S6B). These results extend previous observations obtained in PBMCs with laboratory-adapted HIV-1 NL4-3 V3 recombinants (13). However, exposure to 50% semen led to a substantial accumulation of sperm debris and initial cytotoxicity in the final cell culture concentration of 3.3% semen (vol/vol), as previously shown (13, 23, 24), and was thus excluded from further analysis.

**Fig 4:**
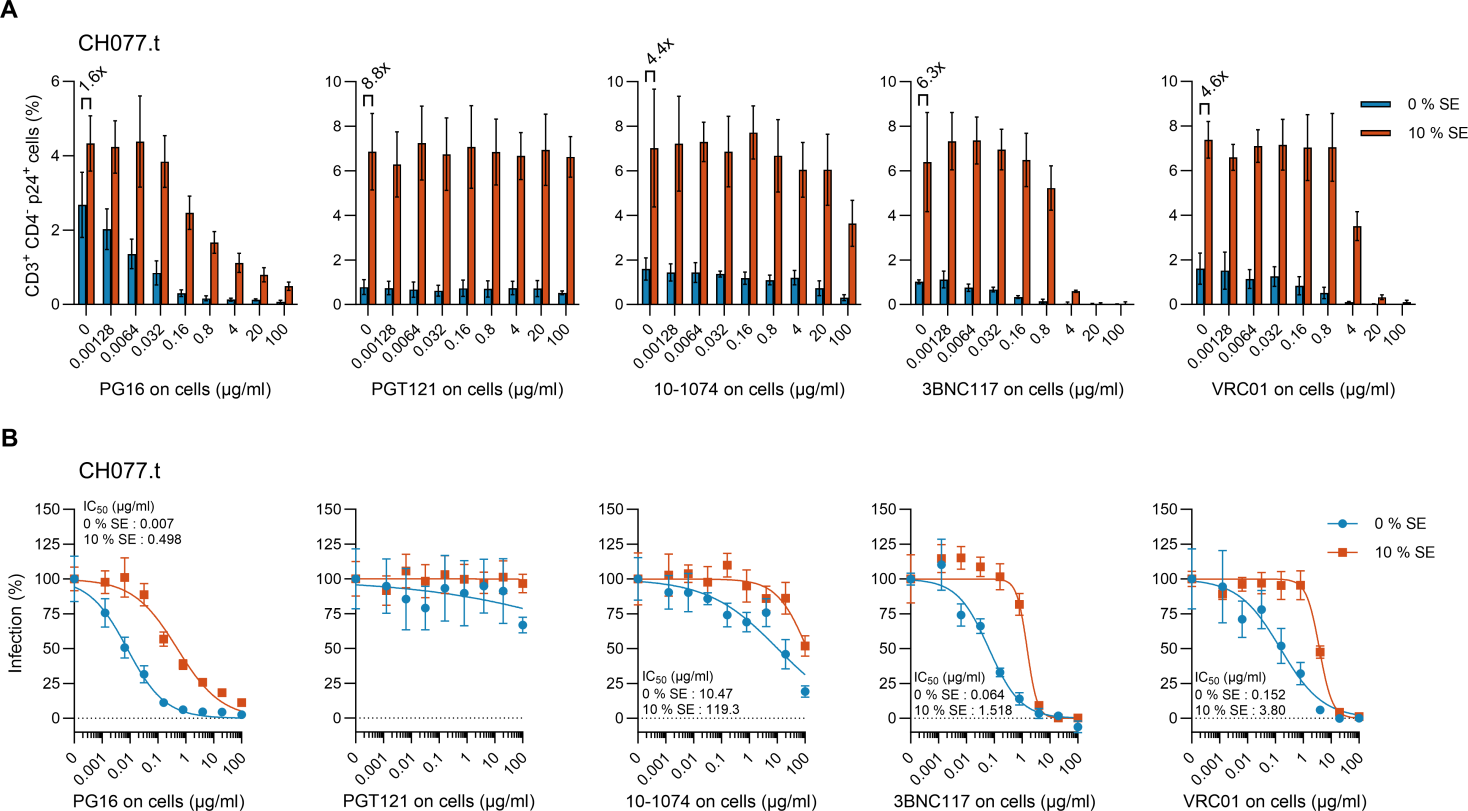
Effect of bNAbs on mock- and semen-exposed HIV-1 CH077.t infection of CD4+ T cells. PBMCs treated with serial dilutions of indicated bNAbs were infected with 10% semen- (10% SE) or buffer (0% SE)-treated HIV-1 T/F strain CH077.t. Infection rates were assessed 3 days later by p24gag staining measured by flow cytometry, normalized to mock-treated cells. (**A**) Infection rates of *n* = 4 individual donors in singlicates ± SD. Numbers above the columns give the n-fold increase in infection of semen- vs mock-treated infection. (**B**) Normalized infection rates of the corresponding graphs in A.

We next evaluated the antiviral effectiveness of bNAbs against both untreated and 10% semen-exposed HIV-1 CH077.t. PBMCs, treated with varying concentrations of bNAbs, were inoculated with either control or semen-exposed virus, and again, infection rates were determined by p24 staining in CD4 target cells (see Fig. 4). Semen exposure resulted in an average increase in the percentage of infected cells by 5.1-fold (Fig. 4A). In the absence of semen, bNAbs PG-16, 10–1074, 3BNC117, and VRC01 impeded CH077.t infection in a dose-dependent manner, showing IC_50_ values of 0.007, 10.47, 0.064, and 0.152 µg/mL, respectively. Conversely, PGT121 was ineffective (Fig. 4A and B). With semen present, the IC_50_ values rose to 0.5, 119, 1.5, and 3.8 µg/mL, respectively. Notably, the more potent antibodies, 3BNC117 and VRC01, maintained their ability to fully block the viral infection of CD4+ T cells, even in the presence of semen. These results in CD4 target cells align with those obtained in TZM-bl cells, indicating the most efficient neutralization of HIV-1 CH077.t by bNAb PG16, followed by 3BNC117, VRC01, and 10–1074, and the absence of neutralization by PGT121.

## DISCUSSION

Semen enhances the infection of HIV-1 and impairs the antiviral efficacy of candidate microbicides, possibly explaining why certain antivirals successfully curb HIV-1 infection *in vitro* but fail to do so in clinical trials (13).

Here, we report that the anti-HIV-1 activities of bNAbs PG16, PGT121, 10–1074, 3BNC117, and VRC01 are less affected by semen-mediated HIV-1 enhancement than that of anionic polymers PSA and CS. This difference might be explained by other modes of inhibition: anionic polymers protect cells in an undirected manner by electrostatic repulsion of negatively charged virus particles, whereas bNAbs specifically target the HIV-1 envelope glycoprotein (16–20, 25). It has been suggested that the alkaline pH of semen reduces the antiviral activity of polyanionic microbicides (26, 27), although it appears unlikely that the final concentration of semen (< 0.7% on cells) is able to neutralize the charge of the polyanions, on top of that in a buffered solution. Cationic semen-amyloids interact with virus particles and bridge the repulsion between the negatively charged cell and virus membrane (8, 10, 28). This leads to an enhanced and accelerated viral attachment, which might also overcome the negative additional repulsion provided by polyanionic microbicides, but not the direct neutralization of HIV-1 mediated by bNAbs. Consequently, the loss of microbicide efficacy is most prominent for negatively charged polyanions while being less pronounced for the bNAbs tested herein, a trend that is also observed in human PBMCs (13).

Interestingly, the bNAbs PG16, PGT121, 10–1074, 3BNC117, and VRC01 are less affected by the presence of semen than the earlier discovered bNAb 2G12 (targeting gp120) and 2F5 (targeting the membrane-proximal external region (MPER) of gp41) (13). This argues that the increased potency of the bNAbs tested herein compared with 2G12 and 2F5 further decreases their susceptibility to semen-mediated enhancement of HIV-1 infection. Taken together, these data suggest that the aforementioned bNAbs are suitable candidates for preclinical development as microbicides. Despite their superior neutralization breadth, certain HIV-1 T/F are resistant toward bNAbs, which is not surprising, given the enormous genetic variety and mutation rates of HIV-1. Thus, this raises the need for administering either combination of bNAbs that target various gp120 epitopes or combinations of bNAbs with antiretrovirals, preferentially host-directed drugs that are effective independently of the presence of semen, such as maraviroc (13).

## MATERIALS AND METHODS

### Cell culture

TZM-bl reporter cells (NIH ARP5011) and HEK293T cells (ATCC) were cultivated in Dulbecco’s Modified Eagle’s Medium (DMEM) supplemented with 10% FCS, 2 mM L-glutamine, 100 U/mL penicillin, and 100 µg/mL streptomycin. For experiments in the presence of semen, media were additionally supplemented with 50 µg/mL gentamycin. PBMCs were isolated from whole blood obtained from the DRK Blutspendezentrum Ulm, and for T-cell activation, it was cultivated in Gibco RPMI 1640 Medium supplemented with 10% FCS, 2 mM L-glutamine, 100 U/mL penicillin, 100 µg/mL streptomycin, 10 ng/mL IL-2, and 1 µg/mL phytohemagglutinin (PHA) for 3 days.

### Semen samples

Liquified semen samples from >20 anonymous donors were obtained from the Kinderwunschzentrum Ulm, approved by the ethics committee of Ulm University, vote number 89/16 - Zo/bal. Samples were pooled and stored at −80°C until usage.

### HIV-1 T/F virus stocks

Infectious molecular clones of HIV-1 T/F IMCs were obtained by the transient transfection of 3.5 million HEK293T cells with 15 µg of respective proviral DNA using TransIT-LT1 Transfection reagent (Mirus) according to the manufacturer’s protocol. Briefly, 45 µL transfection reagent and plasmid DNA were mixed in Opti-MEM serum-reduced medium for 20 min before adding to cells dropwise. After 2 days, virus-containing supernatants were clarified by centrifugation and aliquoted and stored at −80°C. For PBMC experiments, a fresh virus supernatant was 15-fold spin-concentrated through a layer of 20% sucrose in the ratio of 1:5 virus:sucrose at 20,000 × *g* for 3 h. Subsequently, the supernatant was discarded, and the virus pellet was resuspended in supplement-free DMEM.

### Antibody production

Antibodies were produced in HEK293-6E cells (National Research Council Canada) by transfection of expression plasmids for heavy and light chains by using 25 kDa branched polyethylenimine (PEI) (Sigma-Aldrich). Cells were maintained at 37°C and 6% CO2 in FreeStyle 293 Expression Medium (Thermo Fisher) and 0.2% penicillin/streptomycin (Thermo Fisher). Five to seven days after transfection, culture supernatants were harvested, filtered, and incubated with Protein G Sepharose (GE Life Sciences) overnight at 4°C. Antibodies were eluted from chromatography columns using 0.1 M glycine (pH = 3.0) and buffered in 1 M Tris (pH = 8.0). Subsequent buffer exchange to PBS and antibody concentration was performed using Amicon 30 kDa spin membranes (Millipore). Antibodies were filter-sterilized using Ultrafree-CL or Ultrafree-MC 0.22 µm membranes (Millipore) and stored at 4°C.

### Infection of HIV-1 T/F in the presence of semen

Five thousand TZM-bl cells were seeded in 100 µL supplemented DMEM in 96-flat-well plates. The next day, the medium was replaced by 280 µL of supplemented DMEM +50 µg/mL gentamycin. Semen samples were thawed, briefly vortexed, and diluted in PBS. Semen or PBS was mixed 1:1 with 2-fold serial dilutions of HIV-1 T/F IMCs to obtain semen concentrations of 50%, 10%, 2%, and 0.4% during virus incubation. After 5 min incubation at room temperature (RT), TZM-bl cells were infected with 20 µL of virus:semen mixture. At 2 hpi, inocula were removed, cells were washed with PBS, and 200 µL of supplemented DMEM +50 µg/mL gentamycin was added. At 48 hpi, infection rates were measured by assessing β-galactosidase activity in cell lysates using the Gal-Screen system (Applied Biosystems) supplemented with 0.16% Triton-X (Sigma #T9284). β-galactosidase-induced luminescence due to substrate conversion was quantified using an Orion microplate luminometer (Berthold).

### Infection of HIV-1 T/F in the presence of semen in primary cells

In total, 200,000 activated PBMCs were seeded in 280 µL X-VIVO 10 serum-free media (Lonza) supplemented with 2 mM L-glutamine, 100 U/mL penicillin and 100 µg/mL streptomycin +50 µM aminoguanidin, and 50 µg/mL gentamycin. Semen samples were thawed, briefly vortexed, and diluted in PBS. Semen or PBS was mixed 1:1 with 5-fold serial dilutions of HIV-1 T/F IMCs to obtain semen concentrations of 50%, 10%, 2%, and 0% during virus incubation. After 5 min incubation at RT, PBMCs were infected with 20 µL of virus:semen mixture. 3 dpi cells were washed with PBS and stained for flow cytometry measurement. Live/dead stain (Live-or-Dye NucFix Red Staining Kit, Biotium #32010) was diluted in PBS, and cells were stained according to the manufacturer’s instructions. Subsequently, cells were stained for CD3/CD4 surface markers (CD3 Biolegend #317340 /CD4 Biolegend #317434) in PBS supplemented with 1% FCS. Cells were fixed/permeabilized using the BD Cytofix/Cytoperm Fixation/Permeabilization Kit (BD #554714), and infection rates were determined by intracellular p24gag staining (Beckman Coulter #6604665), followed by fixation in 4% PFA, measured at the CytoFLEX LX (Beckman Coulter). The flow cytometry results were analyzed using FlowJo v10.8 Software (BD Life Sciences) with the gating strategy shown in Fig. S5.

### Neutralization activity of bNAbs against HIV-1 T/F

Five thousand TZM-bl cells were seeded in 100 µL supplemented DMEM in 96-flat-well plates. The next day, the medium was replaced by 160 µL of supplemented DMEM. 20 µL of 5-fold serial dilutions of bNAbs in PBS were added to cells. Cells were infected with 20 µL of infectivity-normalized HIV-1 T/F IMCs. Maximum final bNAb concentration on cells was 100 µg/mL. At 48 hpi, infection rates were assessed as described above.

### Neutralization activity of bNAbs and polyanions against semen-exposed HIV-1 T/F

Five thousand TZM-bl cells were seeded in 100 µL supplemented DMEM in 96-flat-well plates. The next day, the medium was replaced by 250 µL of supplemented DMEM +50 µg/mL gentamycin, and 30 µL of 5-fold serial dilutions of bNAbs or polyanions in PBS was added to cells. Semen samples were thawed, briefly vortexed, and diluted in PBS. Semen dilution or PBS was mixed 1:1 with infectivity normalized HIV-1 T/F IMCs, resulting in 10% semen during virion treatment and incubated for 5 min at RT. Cells were infected with 20 µL of semen:virus or semen:PBS mixture. At 2 hpi, inocula were removed, cells were washed with PBS, and 200 µL of supplemented DMEM +50 µg/mL gentamycin +respective bNAb or polyanion was added. Maximum final bNAb or polyanion concentration on cells was 100 µg/mL. At 48 hpi, infection rates were determined as described above.

### Neutralization activity of bNAbs against semen-exposed HIV-1 T/F in primary cells

In total, 200,000 activated PBMCs were seeded in 250 µL X-VIVO 10 serum-free media (Lonza) supplemented with 2 mM L-glutamine, 100 U/mL penicillin, and 100 µg/mL streptomycin +50 µM aminoguanidin and 50 µg/mL gentamycin. Therefore, 30 µL of 5-fold serial dilution of bNAbs in PBS was added to cells. Maximum final bNAb concentration on cells was 100 µg/mL. Semen samples were thawed, briefly vortexed, and diluted in PBS. Semen dilution or PBS was mixed 1:1 with concentrated HIV-1 T/F IMCs, resulting in 10% semen during virion treatment, incubated for 5 min at RT. Cells were infected with 20 µL of semen:virus or semen:PBS mixture. Three days later, cells were stained for flow cytometry measurement as described above.

## Data Availability

All data supporting the findings of this study are available upon reasonable request to the corresponding author.

## SUPPLEMENTAL MATERIAL

Supplemental figures jvi.01190-23-s0001.pdf Figures S1 to S6.

ASM does not own the copyrights to Supplemental Material that may be linked to, or accessed through, an article. The authors have granted ASM a non-exclusive, world-wide license to publish the Supplemental Material files. Please contact the corresponding author directly for reuse.
