# Supplementary material for: Semen enhances transmitted/founder HIV-1 infection and only marginally reduces antiviral activity of broadly neutralizing antibodies
Source: J Virol. 2024 Mar 19;98(4):e01190-23. doi: 10.1128/jvi.01190-23 (PMC11019787; doi:10.1128/jvi.01190-23)
Supplement: Supplemental figures — Figures S1 to S6. [file jvi.01190-23-s0001.pdf]

## Supplemental Figures

# Semen enhances transmitted/founder HIV-1 infection and only marginally reduces antiviral activity of broadly neutralizing antibodies

Pascal von Maltitz[a], Lukas Wettstein[a], Tatjana Weil[a], Philipp Schommers[b,c,d,e], Florian Klein[b,c,d], Jan Münch[a,#]

[a]Institute of Molecular Virology, University Ulm Medical Center, 89081 Ulm, Germany

[b]Laboratory of Experimental Immunology, Institute of Virology, University of Cologne, Faculty of Medicine and University Hospital of Cologne, 50931 Cologne, Germany

[c]German Center for Infection Research, Partner site Bonn-Cologne, 50931 Cologne Germany

[d]Center for Molecular Medicine Cologne (CMMC), 50937, Cologne, Germany

[e]Department I of Internal Medicine, Faculty of Medicine and University Hospital of Cologne, University of Cologne, 50931 Cologne, Germany

## Supplementary Figures:

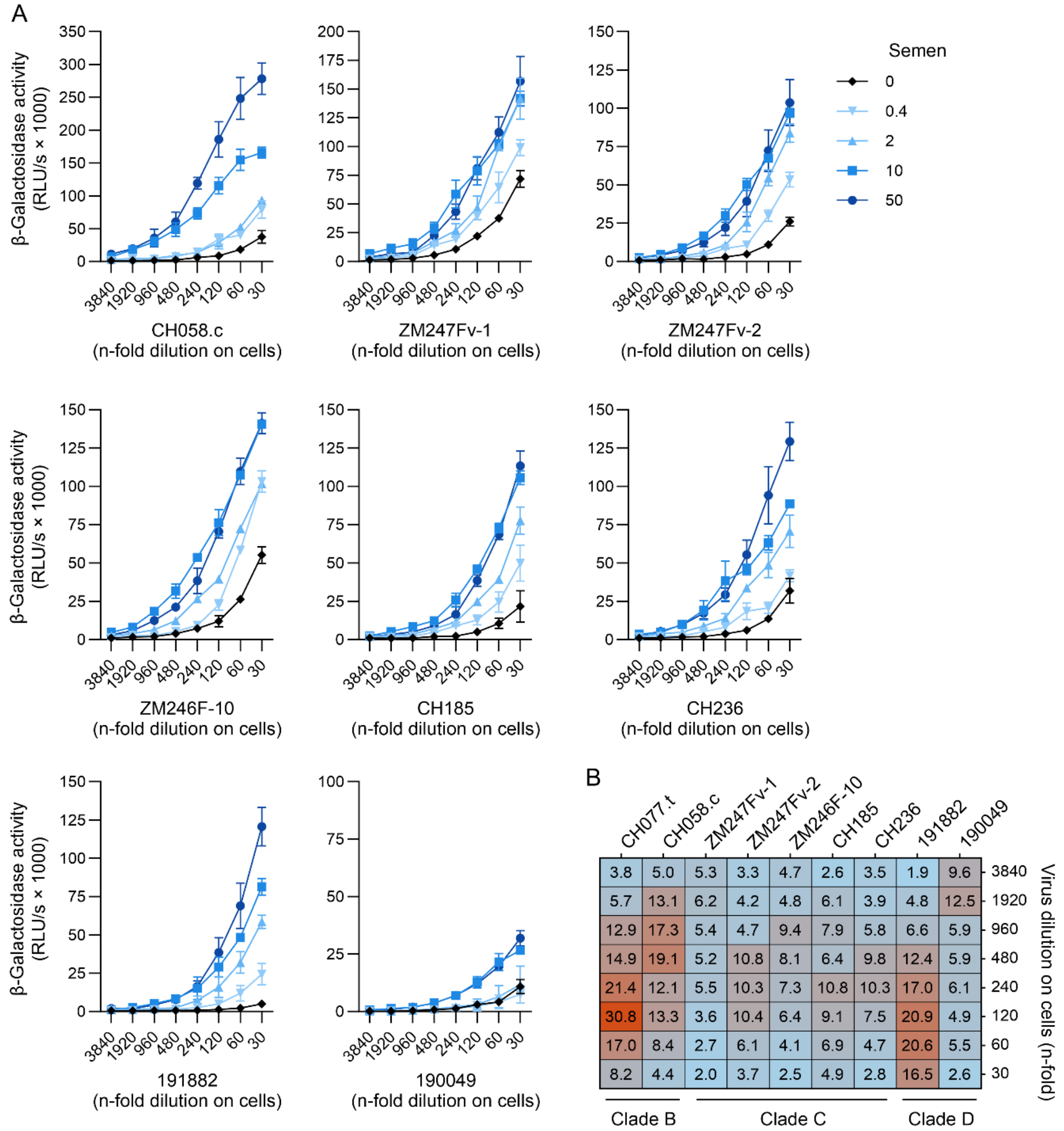


**Fig. S1. Semen enhances HIV-1 transmitted/founder infection.** (A) Infection rates of TZM-bl cells infected with serial dilutions of semen (SE)-exposed (50; 10; 2; 0,4 %) or PBS-treated (0%), replication competent HIV-1 transmitted founder (T/F) strains assessed at 2 dpi by measuring ß-galactosidase activity of cells lysates. (B) Fold infectivity enhancement of respective HIV-1 T/F strains exposed to 10 % SE normalized to infectivity in the absence of semen. Shown are mean values from triplicate infections ± SD.

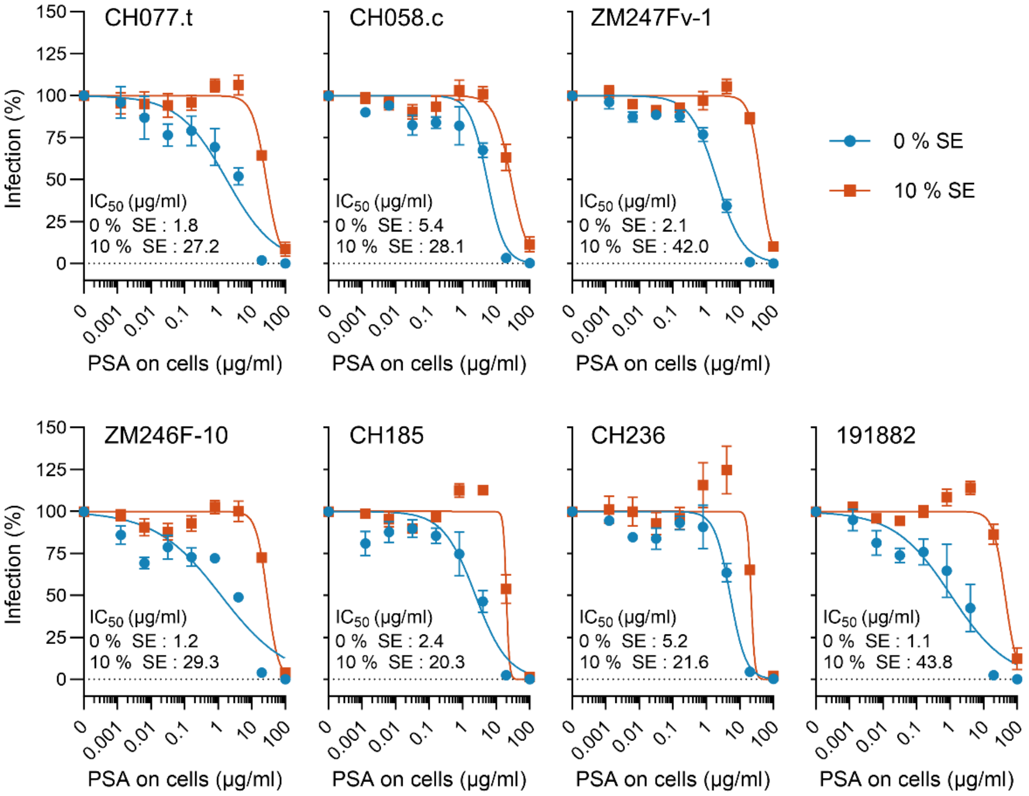


**Fig. S2. Inhibition of HIV-1 transmitted/founder strains by polystyrene sulfonic acid is reduced in presence of semen.** TZM-bl cells treated with serial dilutions of polystyrene sulfonic acid (PSA) and infected with semen- (SE) or PBS-treated, replication competent HIV-1 transmitted/founder (T/F) strains. At 2 hpi, supernatants were removed, cells were washed and fresh medium supplemented with PSA was added. Infection rates were assessed at 2 dpi by measuring ß-galactosidase activity of cell lysates and were normalized to mock-treated cells (n=3 independent experiments, each in triplicates ± SEM).

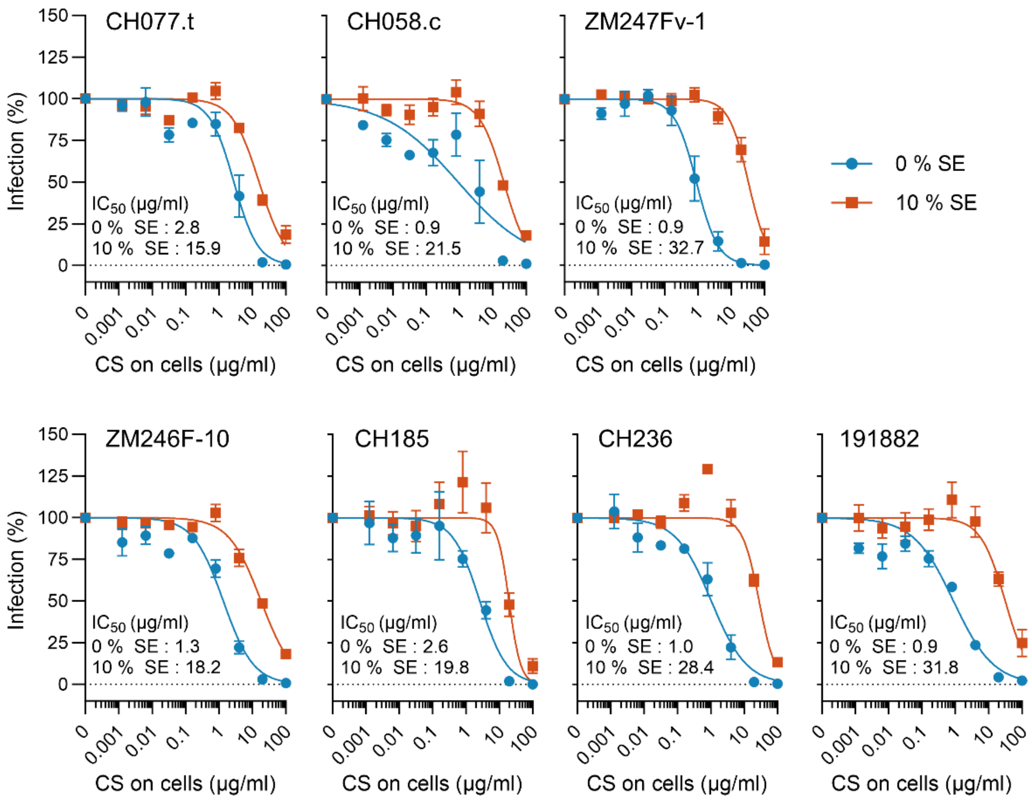


**Fig. S3. Inhibition of HIV-1 transmitted/founder strains by cellulose sulfate is reduced in presence of semen.** TZM-bl cells treated with serial dilutions of cellulose sulfate (CS) and infected with semen- (SE) or PBS-treated, replication competent HIV-1 transmitted/founder (T/F) strains. At 2 hpi, supernatants were removed, cells were washed and fresh medium supplemented with CS was added. Infection rates were assessed at 2 dpi by measuring ß-galactosidase activity of cell lysates and were normalized to mock-treated cells (n=3 independent experiments, each in triplicates ± SEM)

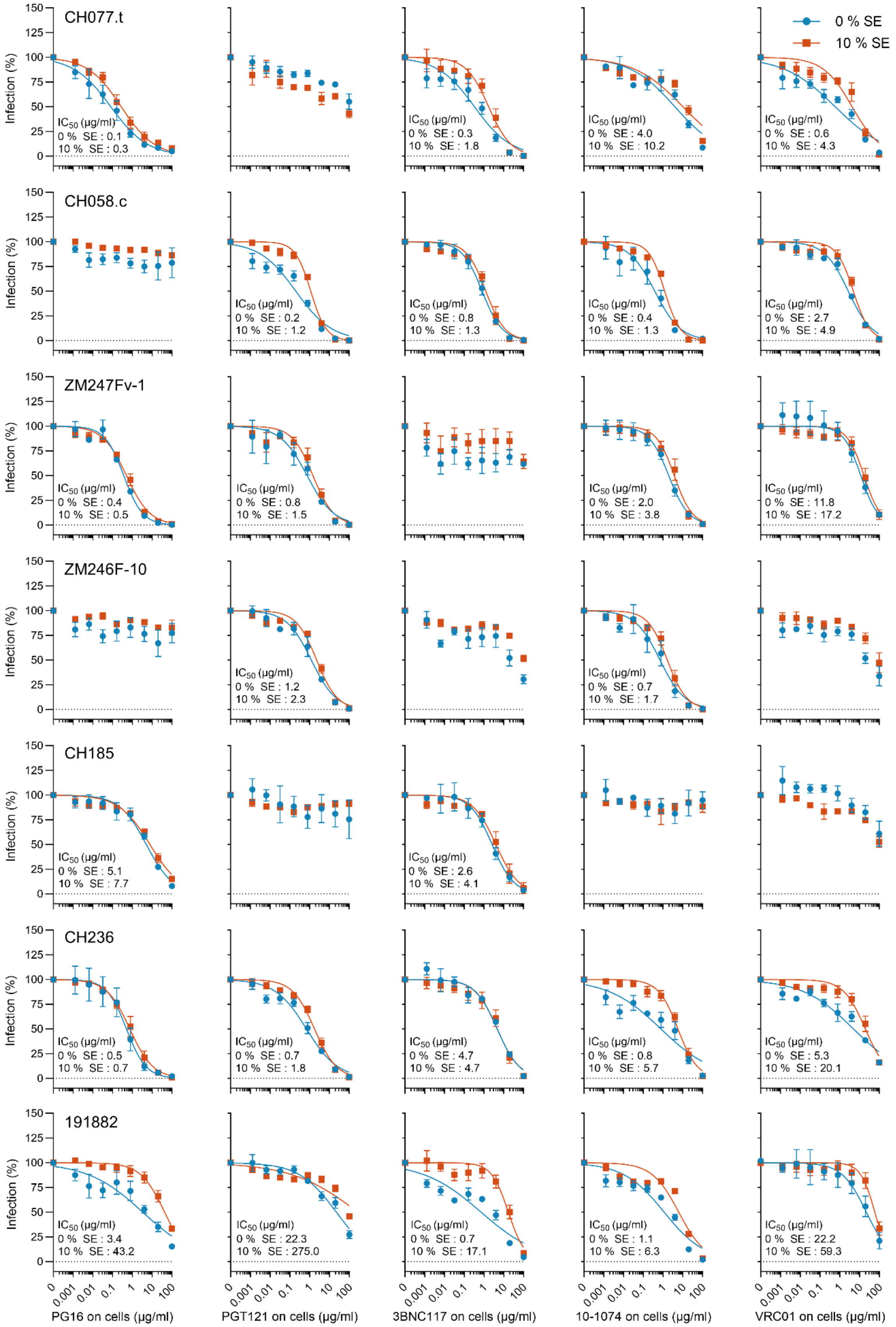

0 % SE
10 % SE
Infection (%)
150
125
100
75
50
25
0
CH077.t
IC50 (µg/ml)
0 % SE : 0.1
10 % SE : 0.3
IC50 (µg/ml)
0 % SE : 0.3
10 % SE : 1.8
IC50 (µg/ml)
0 % SE : 4.0
10 % SE : 10.2
IC50 (µg/ml)
0 % SE : 0.6
10 % SE : 4.3
CH058.c
IC50 (µg/ml)
0 % SE : 0.2
10 % SE : 1.2
IC50 (µg/ml)
0 % SE : 0.8
10 % SE : 1.3
IC50 (µg/ml)
0 % SE : 0.4
10 % SE : 1.3
IC50 (µg/ml)
0 % SE : 2.7
10 % SE : 4.9
ZM247Fv-1
IC50 (µg/ml)
0 % SE : 0.4
10 % SE : 0.5
IC50 (µg/ml)
0 % SE : 0.8
10 % SE : 1.5
IC50 (µg/ml)
0 % SE : 2.0
10 % SE : 3.8
IC50 (µg/ml)
0 % SE : 11.8
10 % SE : 17.2
ZM246F-10
IC50 (µg/ml)
0 % SE : 1.2
10 % SE : 2.3
IC50 (µg/ml)
0 % SE : 0.7
10 % SE : 1.7
CH185
IC50 (µg/ml)
0 % SE : 5.1
10 % SE : 7.7
IC50 (µg/ml)
0 % SE : 2.6
10 % SE : 4.1
CH236
IC50 (µg/ml)
0 % SE : 0.5
10 % SE : 0.7
IC50 (µg/ml)
0 % SE : 0.7
10 % SE : 1.8
IC50 (µg/ml)
0 % SE : 4.7
10 % SE : 4.7
IC50 (µg/ml)
0 % SE : 0.8
10 % SE : 5.7
IC50 (µg/ml)
0 % SE : 5.3
10 % SE : 20.1
191882
IC50 (µg/ml)
0 % SE : 3.4
10 % SE : 43.2
IC50 (µg/ml)
0 % SE : 22.3
10 % SE : 275.0
IC50 (µg/ml)
0 % SE : 0.7
10 % SE : 17.1
IC50 (µg/ml)
0 % SE : 1.1
10 % SE : 6.3
IC50 (µg/ml)
0 % SE : 22.2
10 % SE : 59.3
0
0.001
0.01
0.1
1
10
100
PG16 on cells (µg/ml)
PGT121 on cells (µg/ml)
3BNC117 on cells (µg/ml)
10-1074 on cells (µg/ml)
VRC01 on cells (µg/ml)

**Fig. S4. Inhibition of HIV-1 transmitted/founder strains by bNAbs is moderately reduced in presence of semen.** TZM-bl cells treated with serial dilutions of broadly neutralizing antibodies (bNAb) and infected with respective semen- (SE) or PBS-treated, replication competent HIV-1 transmitted/founder (T/F) strain. At 2 hpi, supernatants were removed, cells were washed and fresh medium supplemented with bNAb was added. Infection rates were assessed at 2 dpi by measuring ß-galactosidase activity of cell lysates and were normalized to mock-treated cells. Normalized Infection rates of n=3 independent experiments, each in triplicates ± SEM.

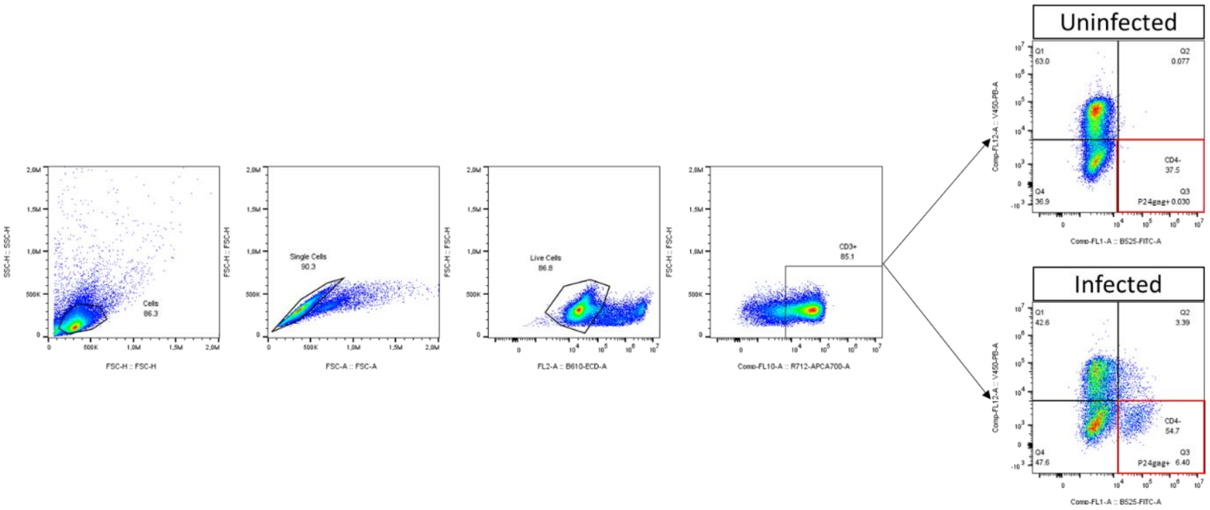


**Fig. S5. Gating strategy of PBMC flow cytometry p24gag measurement.** Cells were gated for single cells; live cells were checked for t-cells by CD3 marker. To ensure that only infected cells are measured CD4- (receptor internalized) cells were checked for infection by intracellular p24gag staining (indicated by red rectangles). Infection in % was extracted from the plots with values for uninfected subtracted as background from all samples tested.

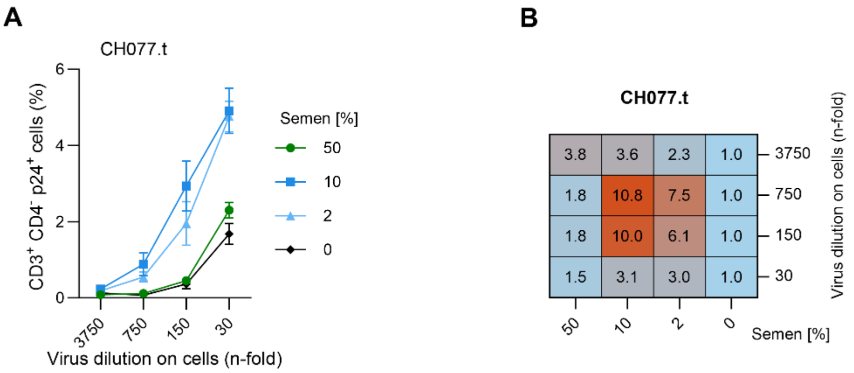


**Fig. S6. Semen enhances HIV-1 transmitted/founder infection in PBMCs.** (A) Infection rates of PBMCs infected with serial dilutions of semen (SE)-exposed (50; 10; 2 %) or PBS-treated (0 %), replication competent HIV-1 transmitted founder (T/F) CH077.t were assessed at 3 dpi by measuring p24gag presence in CD3+/CD4- cells. (B) Fold infectivity enhancement of SE-exposed (50; 10; 2 %) replication competent HIV-1 T/F CH077.t strain normalized to infectivity in the absence of semen. A/B show mean values from n=4 individual donors in singlicates ± SD.
